# Supplementary figures and images for: ST11 Carbapenem-Resistant Klebsiella pneumoniae Clone Harboring blaNDM Replaced a blaKPC Clone in a Tertiary Hospital in China
Source: Antibiotics (Basel). 2022 Oct 7;11(10):1373. doi: 10.3390/antibiotics11101373 (PMC9598860; doi:10.3390/antibiotics11101373)

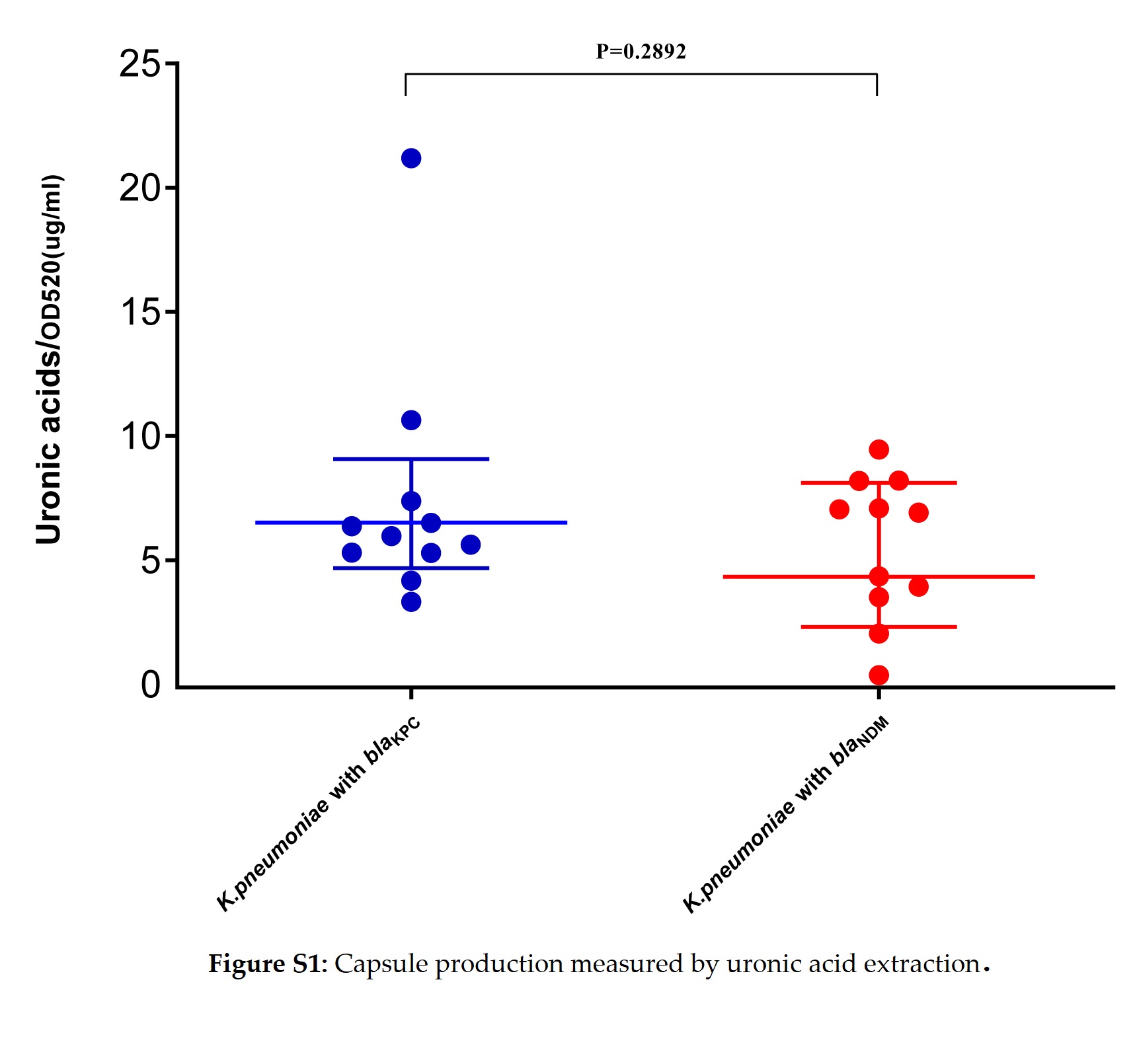

Supplement: Supplementary file 1 [file antibiotics-11-01373-s001.zip › antibiotics-1929496-Figure-S1.jpg]
